# Challenges and Alternatives to Evaluation Methods and Regulation Approaches for Medical Apps as Mobile Medical Devices: International and Multidisciplinary Focus Group Discussion

**DOI:** 10.2196/54814

**Published:** 2024-09-30

**Authors:** Laura Maaß, Robert Hrynyschyn, Martin Lange, Alexandra Löwe, Kathrin Burdenski, Kaley Butten, Sebastian Vorberg, Mariam Hachem, Aldo Gorga, Vittorio Grieco, Vincenzo Restivo, Giuseppe Vella, Marlien Varnfield, Felix Holl

**Affiliations:** 1 University of Bremen, SOCIUM - Research Center on Inequality and Social Policy Department of Health, Long Term Care and Pensions Bremen Germany; 2 Leibniz ScienceCampus Digital Public Health Bremen Germany; 3 Digital Health Section European Public Health Association (EUPHA) Utrecht Netherlands; 4 Charité – Universitätsmedizin Berlin, corporate member of Freie Universität Berlin and Humboldt-Universität zu Berlin, Institute of Health and Nursing Science Institute of Health and Nursing Science Berlin Germany; 5 Department of Fitness & Health IST University of Applied Sciences Düsseldorf Germany; 6 School of Sport, Exercise and Health Sciences Loughborough University Loughborough United Kingdom; 7 Australian eHealth Research Centre (CSIRO) Brisbane Australia; 8 QuR.digital - Vorberg.law Hamburg Germany; 9 Bundesverband Internetmedizin eV Hamburg Germany; 10 Department of Medicine, Austin Health Faculty of Dentistry, Medicine and Health Sciences University of Melbourne Melbourne Australia; 11 Australian Centre for Accelerating Diabetes Innovations Faculty of Dentistry, Medicine and Health Sciences University of Melbourne Melbourne Australia; 12 Department of Sciences of Public Health and Pediatrics University of Turin Turin Italy; 13 Department of Medical, Surgical Sciences and Advanced Technologies “GF Ingrassia” University of Catania Catania Italy; 14 School of Medicine University of Enna Enna Italy; 15 Department of Health Promotion, Maternal and Infant Care, Internal Medicine and Medical Specialties (PROMISE) “G. D’Alessandro” University of Palermo Palermo Italy; 16 DigiHealth Institute Neu-Ulm University of Applied Sciences Neu-Ulm Germany

**Keywords:** medical apps, mobile medical devices, evaluation methods, mobile medical device regulation, focus group study, alternative approaches, logic model, mobile phone

## Abstract

**Background:**

The rapid proliferation of medical apps has transformed the health care landscape by giving patients and health care providers unprecedented access to personalized health information and services. However, concerns regarding the effectiveness and safety of medical apps have raised questions regarding the efficacy of randomized controlled trials (RCTs) in the evaluation of such apps and as a requirement for their regulation as mobile medical devices.

**Objective:**

This study aims to address this issue by investigating alternative methods, apart from RCTs, for evaluating and regulating medical apps.

**Methods:**

Using a qualitative approach, a focus group study with 46 international and multidisciplinary public health experts was conducted at the 17th World Congress on Public Health in May 2023 in Rome, Italy. The group was split into 3 subgroups to gather in-depth insights into alternative approaches for evaluating and regulating medical apps. We conducted a policy analysis on the current regulation of medical apps as mobile medical devices for the 4 most represented countries in the workshop: Italy, Germany, Canada, and Australia. We developed a logic model that combines the evaluation and regulation domains on the basis of these findings.

**Results:**

The focus group discussions explored the strengths and limitations of the current evaluation and regulation methods and identified potential alternatives that could enhance the quality and safety of medical apps. Although RCTs were only explicitly mentioned in the German regulatory system as one of many options, an analysis of chosen evaluation methods for German apps on prescription pointed toward a “scientific reflex” where RCTs are always the chosen evaluation method. However, this method has substantial limitations when used to evaluate digital interventions such as medical apps. Comparable results were observed during the focus group discussions, where participants expressed similar experiences with their own evaluation approaches. In addition, the participants highlighted numerous alternatives to RCTs. These alternatives can be used at different points during the life cycle of a digital intervention to assess its efficacy and potential harm to users.

**Conclusions:**

It is crucial to recognize that unlike analog tools, digital interventions constantly evolve, posing challenges to inflexible evaluation methods such as RCTs. Potential risks include high dropout rates, decreased adherence, and nonsignificant results. However, existing regulations do not explicitly advocate for other evaluation methodologies. Our research highlighted the necessity of overcoming the gap between regulatory demands to demonstrate safety and efficacy of medical apps and evolving scientific practices, ensuring that digital health innovation is evaluated and regulated in a way that considers the unique characteristics of mobile medical devices.

## Introduction

### Background

**Figure 1:**
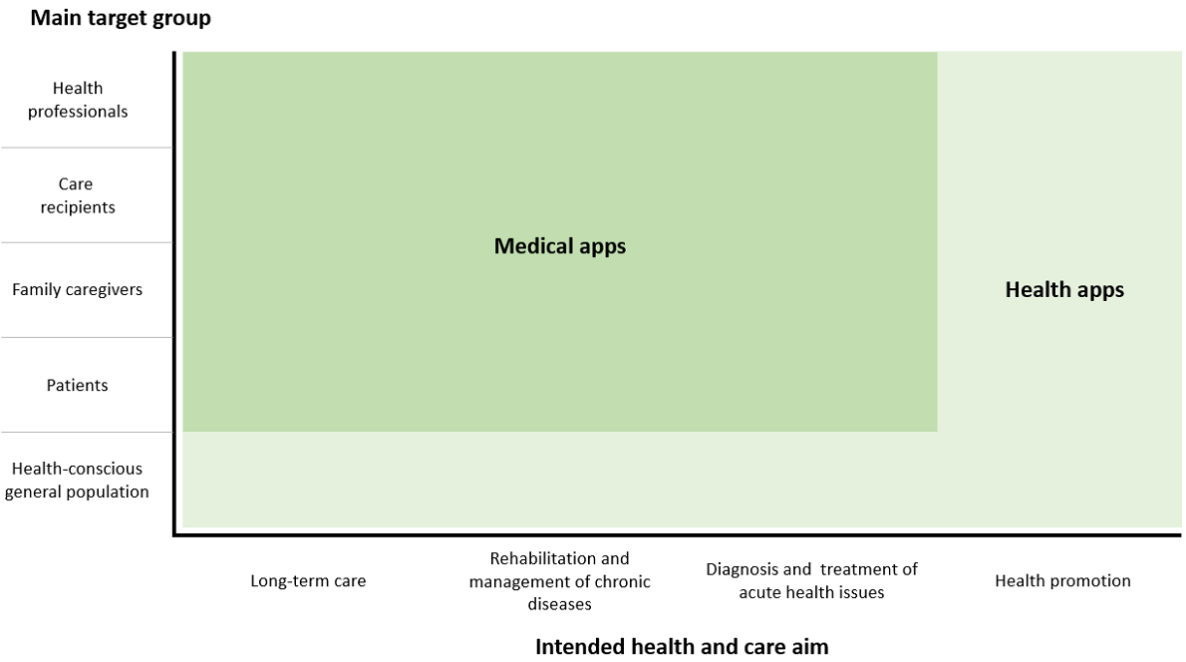
Medical apps as a subgroup of health apps; adapted from Maaß et al [6].

Medical apps have emerged as a subgroup of health apps. They are considered as powerful tools with the potential to revolutionize health care by supporting, managing, and enhancing individual and population health [1]. These software programs can run on mobile devices and offer new possibilities for processing health-related data [2,3]. According to the General Data Protection Regulation, data processing includes any operation performed on personal (health) data, including collecting, organizing, storing, adapting, visualizing, retrieving, disseminating, restricting, or erasing the data [4,5]. Unlike most types of health apps, medical apps do not primarily target healthy or health-conscious individuals but patients, health professionals, or family caregivers. This stems from their primary use as treatment supplements or medical devices in clinical contexts such as for diagnostics, treatment, or rehabilitation. Figure 1 summarizes the distinction between medical apps as a subgroup and other health apps (such as wellness apps) from a legal point of view [6].

Medical apps promise personalized health care, improving access to medical resources and fostering active patient participation [7]. However, this thematic orientation of medical apps introduces a range of potential risks. Health and patient information may be inaccurate or flawed [8]; sensitive data can be disclosed to the wrong recipients or lost [9]; applications frequently target broad user adoption via the internet, increasing the risk of rapid dissemination of potential dangers. Due to the sensitive nature of health data, medical apps must adhere to rigorous data security standards. As many apps are used across borders, different jurisdictions and regulatory standards may need to be met [10,11].

While considering covering the costs of these products through various entities, such as through health insurance providers, demonstrating their clinical and medical benefits becomes essential for them to be regulated as mobile medical devices [12-15]. This advantage must be substantiated and evaluated to align costs adequately within the insurance community [16]. In addition, high data protection requirements, such as General Data Protection Regulation, impact the possibilities of data collection, analysis, and, therefore, the evaluation of medical apps [17-19]. Software is an increasingly critical area of health care product development; there is a need to establish a standard and converged understanding of clinical evaluation globally [20].

### Challenges in the Evaluation of Medical Apps

**Figure 2:**
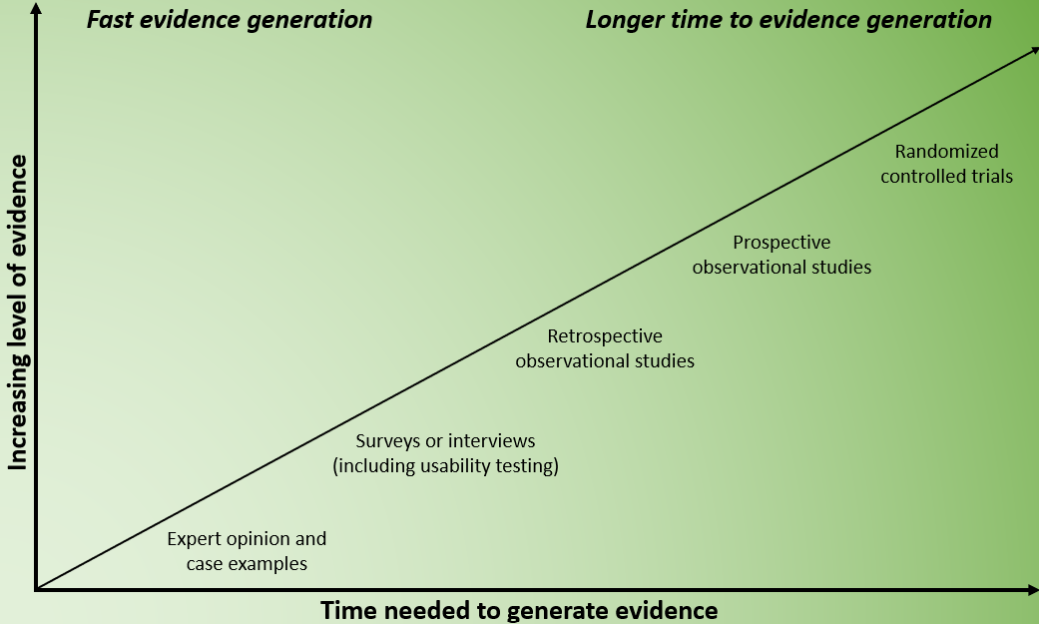
The balancing act between increasing evidence levels and the time needed to generate them; adapted from Guo et al [26].

With the increase in the use of medical apps in health care, it is crucial to validate their effectiveness. Randomized controlled trials (RCTs) are commonly considered the gold standard for establishing the cause-and-effect relationships because of the rigorous methodology used for such trials [21-23]. However, there is an apparent disparity between the design of RCTs and the need to evaluate medical apps: RCTs are structured and rigid, whereas medical apps are versatile, updated frequently, and depend on specific contexts [24]. A critical challenge arises from the discrepancy between the thoroughness of evaluations and the pace of generating evidence, as depicted in Figure 2 [25]. This misalignment underscores the need for developing more adaptable evaluation strategies in the rapidly evolving field of medical technology.

Furthermore, traditional trials such as RCTs do not necessarily align with the unique characteristics of medical apps [27,28]. The evaluations of medical apps is made on the basis of short-term studies [29-31], limited patient samples [32], feasibility trials, and user preference surveys [33]. While these methods can swiftly provide valuable insights during the development phase, they only offer a narrow view of the true efficacy of the apps [34].

Another challenge is that in contrast to the intergroup design of RCTs, medical apps are often customized to individual needs. Continuous updates and tweaks to medical apps aimed toward improving their performance can sometimes shift their fundamental functionality. It is essential to recognize that these updates might influence patient outcomes and call for continuous reassessment [24]. Given users’ diverse health needs, preferences, and technological expertise, a universal evaluation method seems impractical. Moreover, blinding participants and researchers, a cornerstone of RCTs to reduce bias, can be difficult with medical apps. This is because users are often aware of their app use, which will lead to developers struggling to create “placebo” apps with identical functionalities.

In addition, app evaluation is multifaceted, demanding attention to factors beyond the health metrics typically assessed in RCTs [34]. Aspects, such as user engagement and sustained use, are vital for long-lasting behavioral shifts [35-37]. During the developmental phase of a medical app, alternative study designs, including feasibility studies and user preference surveys, might be more suitable [33], even if they offer limited efficacy data [32]. They can promptly provide essential evidence regarding developmental and application areas. The multifaceted demands of medical app evaluation call for innovative evaluation methods that form the foundation of medical app regulations.

### The Complexity and Struggles of Overregulation

Regulatory and quality standards that are legally mandated and government-reviewed ensure medical the safety, efficacy, and data privacy of medical apps [26]. Historically, pharmaceuticals and medical devices have faced increased regulation only after substantial scandals and incidents have emerged. “Fueled” by these scandals, medical app regulation seeks to prevent scandals and incidents through rigorous guidelines [38-40]. However, there is a risk that anticipatory regulation may overemphasize securing against hypothetical risks [41]. It is crucial to weigh hypothetical risks against the effort required for regulatory compliance and the expected benefits of maintaining a balanced regulatory approach [42]. Relevant criteria for regulating medical apps are displayed in Textbox 1 (adapted from Torous et al [43] and Meskó and deBronkart [44]). Regulatory solutions should adopt the mildest measures possible to minimize resistance to technological advancement. Consequently, it is essential to incorporate the potential severity of harm into regulatory assessments and, if necessary, accept manageable errors as learning opportunities. Postmarket surveillance is a critical component of quality control and assurance in the regulation of innovative medical developments.

**Relevant criteria for regulating mobile apps as mobile medical devices.**

**Criteria**

- Effectiveness and safety: Regulatory frameworks should be adapted to the specific characteristics of digital health applications (DiGAs), necessitating clinical studies and testing procedures tailored for these apps.
- Data privacy and security: Regulation must ensure that the applications implement appropriate data protection and security measures.
- Interoperability: DiGAs should be integrable into existing health care systems to ensure seamless care delivery.
- Provider qualifications: Regulations should mandate that qualified professionals develop and operate applications to ensure patient safety and quality.
- Monitoring and traceability: Regulatory mechanisms should enable the ongoing monitoring and traceability of DiGAs to identify and address adverse events promptly.
- Patient involvement: Patients should be actively engaged in the development and evaluation of DiGAs to ensure that their needs and expectations are met.

### Need for Alternative Evaluation Approaches and Regulation

Although the results are prone to reduction and distortion, regulatory requirements homogenize research conditions. At the same time, medical apps are, by nature, complex interventions due to their structure, a large number of interactions, and various intervention components. The efficacy of medical apps depends on context and leads to distinct effects on individuals. This requires a broader evaluation framework that does not focus solely on specific intervention outcomes but combines different perspectives from feasibility testing to intervention, implementation, and impact evaluation [26,45,46]. Current regulatory approaches to evaluating medical apps do not appropriately reflect the complex nature of such apps.

### Regulation of Medical Apps in Selected Countries

To illustrate similarities and differences, 4 countries have been selected to conduct case studies about the existing regulation methods for the use of medical apps as mobile medical devices. The analysis for Germany, Italy, Australia, and Canada followed an adapted version of the policy benchmarking framework by Essén et al [38] (Table 1).

**Table 1:** Overview of medical app regulation for selected countries.

	Germany	Italy	Australia	Canada
Actors developing national policy regulation	Health Innovation Hub, an external, interdisciplinary expert think tank to the Federal Ministry of Health and the Federal Institute for Drugs and Medical Devices	Ministry of Health overseeing conformity following the European Medical Device Regulation and classification of SaMD^a^	TGA^b^	Health Canada (federal government agency)
Intended use of the framework	It helps in determining the eligibility of an app for reimbursement through the public insurance scheme either permanently or on a preliminary basis.	—^c^	It provides national guidance and a helpful reference tool for app developers working on mobile health apps for release in Australia. It provides a solid basis for further research and analysis.	It helps in determining whether the app meets the legal definition of a SaMD.
Key regulations underpinning the policy framework	DiGAs^d^ under the Digital Care Act	—	The Privacy Act 1988, overseen by the Office of the Australian Information Commissioner; the Competition and Consumer Act 2010 administered by the Australian Competition and Consumer Commission	Risk classification as an SaMD
Risk classification framework for medical apps	European Medical Device Regulation (Risk classes I, IIa, IIb and, III)	European Medical Device Regulation (risk classes I, IIa, IIb, and III)	TGA (Medical Devices) Regulation 2002 (risk classes I, IIa, IIb, and III)	Health Canada Medical Device Classification (risk classes I, II, III, and IV)
Reimbursement approval policy regulation developed?	Applications get approved through the DiGA fast-track procedure. Approved DiGAs are reimbursed through health insurance	—	—	—
End-user interface to clinical practice and patients, which lists approved medical apps	DiGA directory	—	—	None. However, Health Canada does engage with the Canadian Agency for Drugs and Technologies to provide evidence and publicly available health technology assessments.
Are RCTs explicitly mentioned in the regulation?	Mentioned as an example of a methodology designed to demonstrate a positive care effect	—	—	—

^a^SaMD: software as a medical device.

^b^TGA: Therapeutic Goods Administration.

^c^Not applicable.

^d^DiGAs: digital health applications.

### Regulations in Germany

Since December 2019, digital health applications (DiGAs) have become eligible for reimbursement through statutory health insurance in Germany. This mechanism is included in the Digital Care Act (DVG) [47,48]. Reimbursable DiGAs are listed in a directory administered by the Federal Institute for Drugs and Medical Devices, DiGA directory. Applications can be added to the directory through a fast-track procedure [49]. Applications can either be permanently included in the list if evidence of a positive care effect is provided at the time of application or a provisional reimbursability is granted when apps meet a set of essential requirements with evidence of the positive care effect is still missing. For permanent inclusion, evidence of the positive care effect must be provided within 12 months. Otherwise, the app will be removed from the list. The term “positive care effect” introduced by the DVG can either be a “medical benefit” or “patient-relevant improvement of structure and processes.” For instance, the app can help improve health literacy and improve coordination of treatment processes or can reduce therapy costs. Currently, most DiGAs provide evidence for the positive care effect using data from RCTs [50]. As of October 13, 2023, the DiGA directory included 55 applications of which 23 were provisionally included, 26 were permanently included, and 6 were removed. Although the DVG does not explicitly call for an RCT [51], all the apps were assessed through an RCT. A limitation of the DVG is the restriction on low-risk devices (risk classes I and IIa) under the Medical Devices Act. All applications outside the Medical Devices Act, such as preventive applications, and high-risk medical devices do not fall under the DVG. The fast-track procedure applies only to applications classified and certified as medical devices in risk classes I and IIa; therefore, many potentially helpful applications do not fall within the scope of the DVG [52].

### Regulations in Italy

In Italy, medical app, provided they meet legal requirements, can be promptly marketed and are classified as software as a medical device (SaMD), and obtain a CE marking [53,54]. On May 25, 2021, the Italian Ministry of Health issued a newsletter to offer interested stakeholders with recommendations for clinical investigations of medical devices in line with current regulations [55]. The Ministry is currently responsible for the approval process. However, there is no national framework for market access or reimbursement approval; a law for the health technology assessment of medical apps is also lacking. While an app can be sold, it cannot be prescribed or reimbursed, hindering equitable health care access. Discussions are ongoing in legislative bodies to address this issue; a Parliamentary Intergroup for Digital Health and Digital Therapies was established in May 2023 [56]. This initiative aims to develop regulations that align with those of other European countries, ideally, during the current legislative term.

### Regulations in Australia

In Australia, medical devices, including software-based medical devices, are classified according to the medical device classification rules of the Therapeutic Goods (Medical Devices) Regulations 2002 [57]. This act oversees the quality, safety, efficacy, and availability of therapeutic goods in Australia. Medical devices are classified and regulated according to the level of harm they may pose to the users or patients. This includes medical device software and medical devices that incorporate software. For the manufacturer to determine the inclusion of an app in the Australian Register of Therapeutic Goods [58], a 4-tiered classification system is used. This is done to determine the minimum conformity assessment procedures or evidence requirements for comparable overseas regulators. The classification rules are applied according to the manufacturer’s intended purpose and the device’s functionality. Where more than 1 rule applies, the device must be classified at the highest applicable level [57,59]. However, the Australian regulatory system lacks refinement regarding emerging technologies such as artificial intelligence in medical decision-making and wearable technology. This generates problems in deciding which tools and devices must be registered and to which classification standard [60].

### Regulations in Canada

Similar to Australia, within Canada, mobile health apps are classified as SaMD and regulated according to a 4-tier risk-based classification system used for medical devices. The manufacturer’s intended use determines the classification of potential SaMD and corresponding regulation. It also determines whether the federal department, Health Canada, agrees with the use case [61]. The clinical and evaluation evidence required to support the use case varies depending on the tiered classification and intended use. However, the guidance on SaMD, released by Health Canada in 2019, suggests broad exclusion criteria, eliminating many health apps from requiring SaMD status, thereby leaving many apps unregulated and without evaluation [62].

### Study Aim

Due to the described challenges in regulating and evaluating medical apps, this study pursued 2 central objectives. Building on the previously described examples of country-specific uses for the current regulation and evaluation practices of medical apps, this study first aimed to determine whether there are international challenges in regulation and evaluation. Second, this study aimed to gather the opinions and perspectives of digital (public) health researchers on current practices and future developments through a focus group approach. The results from both the objectives were then used to develop a logic model proposing a differentiated approach to regulating and evaluating medical apps. Therefore, this study attempts to initiate a discussion about current regulation and evaluation practices of medical apps and accelerate the adoption of innovations in health care systems.

## Methods

### Quality Assessment

The reporting quality of this study is assessed through COREQ (Consolidated Criteria for Reporting Qualitative Research) checklist (Multimedia Appendix 1).

### Focus Group Discussion Workshop at the 17th World Congress on Public Health

Participants for the focus groups were recruited through a convenient sampling method. Interested attendees who are members of the 17th World Congress on Public Health (WCPH) attended the workshop in person. The study was advertised to participants through the conference program. Due to the nature of the context, information on nonparticipation was not recorded. In total, 46 WCPH delegates from 14 countries joined the workshop. Besides the moderators and workshop participants, 2 conference technical-team members were in the room to oversee the technical equipment. They did not participate in the workshop. At the beginning of the session, the participants were introduced to the general concepts of health and medical apps and the problems that RCTs pose for evaluating these types of digital health interventions.

To collect demographic data and information regarding the participants’ knowledge, we used a QR code leading to a LimeSurvey questionnaire (LimeSurvey GmbH) hosted by the University of Bremen (Germany). All questions were optional. The 46 participants were equally distributed among 3 focus groups, with each group led and protocolled by one of the authors. Furthermore, 1 female and 2 male authors introduced themselves as researchers interested in evaluating digital apps and explained the scope of the workshop. They discussed the 2 questions presented in the aim section of the study. The 2 questions were not pilot tested. The 3 authors have a background in public health and medical informatics and have conducted previous research on the topic; one author is pursuing his PhD on alternative evaluation approaches [63]; the second author has published a review on the definitions for health and medical apps [6]; the third author has conducted an assessment of evaluation methods for mobile health interventions [35]. The authors had the underlying assumption that most participants are from Italy and that the discussions will focus more on the first question due to the conference’s target group of public health researchers and practitioners.

Focus groups are used in qualitative research to gain in-depth knowledge of specific issues, which, in our case, is the evaluation and regulation of medical apps. Unlike in surveys or individual interviews, a strength of focus groups is that participants can interact with other participants and the moderator rather than merely sharing their views [64]. In our case, we followed the methodology proposed by Traynor [64] with a minor adaptation to avoid audio recording the discussions due to the noise in the room. Instead, we asked 1 participant per group to assist the moderator in taking notes on a flip chart visible to the respective focus group. The discussion lasted 40 minutes, with the first half of the session focusing on question 1 and the second half on question 2. The moderators asked the 2 guiding questions and follow-up collective questions, encouraged quieter participants to express their opinions, and quietened more dominant speakers if necessary. After the workshop, all participants were invited to contribute to the project as coauthors; following this, 9 participants contributed to the project. The workshop transcripts were only provided to the 9 coauthoring participants (Multimedia Appendix 2).

### Ethical Considerations

The study was conducted in accordance with the Declaration of Helsinki; the protocol was approved by the Joint Ethics Committee of the Universities of Applied Sciences of Bavaria (GEHBa-202304-V-105, dated April 28, 2023). After the introductory presentations described above, the researchers handed out written participant information sheets. This was done to educate the participants on the study and to determine which data would be collected, stored, and analyzed. All data were anonymized. This means that the researchers did not collect information that would allow the identification of participants. All participants were informed that their study participation was voluntary. This means that they could withdraw from the study at any time without providing reasons. Before the data collection process, participants were informed that they would not receive any financial compensation for participating in this study. However, they were invited as coauthors if they expressed written interest. In this case, the participants’ email addresses were collected on a paper sheet. These were not linked to the web survey through which their sociodemographic data were collected. Finally, all participants provided their written informed consent before data collection.

### Qualitative Analysis and Data Synthesis

For the thematic analysis (TA) methodology of Braun and Clarke [65,66], data analysis and synthesis followed the conceptual and design thinking. TA aims to indicate and analyze patterns (so-called themes) in minimally organized data sets. TA was deemed the most suitable framework because it can be applied without preexisting theoretical frameworks. This makes it an ideal methodology for multidisciplinary and interdisciplinary research questions such as the current topics currently discussed in the study [65,66]. In our case, 2 authors individually clustered the workshop results inductively, without predefined themes, in 2 separate Microsoft Excel (Microsoft Corp) sheets. Both authors were provided with transcripts of all the flip charts for the focus groups. They then merged individual statements that were based on the same typology and meaning. For example, when 2 groups mentioned the same evaluation designs or barriers, these were merged together. The results were then grouped according to a shared umbrella theme. The grouping was performed separately for the evaluation and regulation of medical apps. The final themes were approved during a discourse among 3 authors, with 1 author having the ultimate decision-making power.

On the basis of the workshop findings and policy analysis, we developed a logic model to capture the complexity of evaluating and regulating medical apps. Logic models can be helpful to display the life cycle of a medical app based on overarching themes, for instance, problem, target, intervention and content, moderating and mediating factors, outcomes, and impact [67]. These models have been used to evaluate digital health interventions [68,69]. The models can also be adapted to the specific contexts. It enables the display of the interaction and feedback loops. It also distinguishes between outcomes and impacts in different domains [67]. Furthermore, adjusted logic models can provide a framework to display how complex interventions work across multiple domains in a single setting, with interlinking actions producing a range of outputs and outcomes. In our logic model, we used the example of a generic medical app to display substantial findings and assign them to specific phases of the medical app life cycle such as development, implementation, and impact.

## Results

### Overview

In total, 46 experts participated in the 1-hour international workshop at the 17th WCPH [70]. The participants were divided into 3 focus groups of 15 to 16 people each. The participant demographics are displayed in Table 2.

We asked the participants about their experiences and opinions regarding evaluating and regulating medical mobile apps. The TA of the contributions in the 3 groups resulted in 4 clusters for the evaluation and 3 for the regulation of medical mobile apps. These are presented in more detail in Textboxes 2 and 3.

**Table 2:** World Congress on Public Health workshop: participant demographics.

Criteria		Participants (N=46), n (%)
**Highest qualification**		
	Bachelor’s degree	2 (4.3)
	Master’s degree	13 (28.3)
	Diploma	2 (4.3)
	Medical doctor	18 (39.1)
	PhD	11 (23.9)
**Primary discipline**		
	Computer science	5 (10.9)
	Medicine	3 (6.5)
	Psychology	3 (6.5)
	Public health	34 (73.9)
	Sociology	1 (2.2)
**Gender**		
	Women	26 (56.5)
	Men	20 (43.5)
**Country of residence**		
	Australia	5 (10.9)
	Austria	1 (2.2)
	United States	4 (8.7)
	Canada	1 (2.2)
	Finland	8 (17.4)
	Germany	1 (2.2)
	Hungary	1 (2.2)
	Indonesia	17 (37)
	Italy	1 (2.2)
	Netherlands	2 (4.3)
	New Zealand	1 (2.2)
	Philippines	1 (2.2)
	Portugal	1 (2.2)
	Switzerland	1 (2.2)
	Taiwan	1 (2.2)
**Age**		
	Mean (SD; range)	35.1 (9.6; 25-73)
	Median (IQR)	31.5 (8.3)
**Years of general work experience in the primary field**		
	Mean (SD; range)	8.3 (8.5; 1-40)
	Median (IQR)	5 (7.0)
**Years of experience in developing, implementing, regulating, or evaluating health or medical apps**		
	Mean (SD; range)	1.8 (2,7; 0-15)
	Median (IQR)	1 (2.0)

**Results of thematic data analysis on alternative evaluation methods.**

**Traditional randomized controlled trials (RCTs)**

- These determine the strengths of RCTs (eg, internal validity and the strength of causal relationships).
- These determine the weaknesses of RCTs to represent reality, resource consumption, and low generalizability.
- Randomization might cause dissatisfaction and increase in dropouts.
- Effectiveness trials tend to produce too little engagement.
- The speed of app development does not align with RCT duration.

**General evaluation aspects**

- Different types of outcomes need to be acknowledged by evaluation goals and methods.
- Power (ie, a priori sample size and bias) and data validity (ie, level of causality and purity of data) are evaluated.
- Differentiation of traditional (eg, effectiveness and satisfaction) and implementation outcomes (eg, acceptability, costs, and feasibility) is conducted.
- Qualitative prestudy generates fast evidence.
- Levels of engagement and interaction (eg, user frequency and duration of use) are determined.
- Importance of multiple and standardized outcomes is determined.
- A suitable comparison group (ie, new, existing, or nontreatment intervention) is identified.
- Intervention effects (eg, Hawthorne) are considered.
- Outcomes for stakeholder and target groups differ and need to be considered.
- Relevance of the component’s effectiveness is determined.
- Relevance of output evaluation is determined.

**Alternatives for recruiting and grouping in RCTs**

- Preference-based controlled trials
- Evaluation of specific implementation stages rather than considering implementation as a whole
- Pragmatic RCTs or real-world RCTs
- Ensuring data quality through volunteer participation
- Quasi-controlled trial
- Best-choice experiment after randomization
- Stepped wedge trials
- Waitlist-control-group design
- Evaluation as treated

**Alternatives to RCTs**

- Collecting qualitative empirical data (eg, user interviews) to understand engagement
- Improving user experience to promote engagement
- Creating a prototype evaluation checklist for researchers with less experience in app development.
- Using real-world evidence (eg, hospital data and cohort data set)
- Using shortcut evaluation if a known aspect works (eg, McCarthy Evaluation)
- Evaluating the practice or rollout phase
- Using model apps through existing, similar data sets
- Applying user analytics (eg, Google Analytics)
- Using of additional data through smartphone sensors

**Results of thematic data analysis on alternative regulation approaches.**

**Functionality of medical apps**

- Regulation should be proportional to app functionality.
- No discrimination and not doing any harm need to be considered as the minimum requirements for the analysis.
- Privacy of data needs to be ensured.
- Accountability and informed consent are considered as the developer’s responsibility.
- Efficacy should be measured.

**Tools for regulation**

- Using labels that rate various aspects of the app (eg, accessibility and health benefits)
- Standardizing regulation across geographical areas (eg, in Europe, create minimal requirements)

**Barriers to regulation**

- There should be no overregulation.
- Silo apps: if a chronically person needs 20 apps to manage their symptoms, this is unfeasible and expensive.
- It is equally important to consider the device as the app.

### Alternative Evaluation Methods

The following general question was presented to the participants at the beginning of the focus group discussions: What could be the alternative evaluation methods for assessing the effectiveness of medical applications? According to the participants, 4 overarching themes were crystallized as pivotal (Textbox 2).

#### Traditional Evaluation Practice

Participants emphasized the advantages of RCTs in evaluating medical apps, acknowledging their ability to reduce bias and establish causal relationships. However, concerns were raised about participant dissatisfaction and high attrition rates due to random assignment, questioning sustainable implementation and user centricity. Incongruence between the temporal requirements of an RCT and the rapid evolution of medical apps necessitated frequent updates, prompting the consideration of alternative evaluation methods.

#### Universal Considerations for All Evaluation Types

The importance of engagement and user data in app evaluations was highlighted; participants advocated qualitative assessment in prestudy designs to reduce dropouts. In addition, the participants stated that standard outcome parameters were essential for effectiveness evaluation and could be supplemented by output data to demonstrate efficacy. The participants further suggested segmental app evaluations and various comparative interventions. Regardless of the study design, the importance of enhancing power and data quality through a priori sample calculations and suitable study designs was highlighted.

#### Alternative Approaches for Participant Recruitment and Grouping in an RCT

Alternative recruitment and grouping methods were explored to address dissatisfaction and attrition among the participants of RCTs. These included preference clinical trials, pragmatic RCTs, partially randomized patient-preference trials, and best-choice experiments. By increasing flexibility and allowing participants to choose their interventions, these approaches might reduce withdrawal, shorten study duration, reduce bias, and facilitate participant recruitment.

#### Alternative Research Designs to RCTs

Other research designs, such as qualitative and participatory approaches, can help to understand engagement barriers and enhance user experience. Prototype checklists were proposed for researchers unfamiliar with alternative evaluation designs. Alternative data sources, including real-world evidence, smartphone sensors, and commercially available user analytics, can complement traditional evaluation methods.

A glossary explaining the alternative evaluation methods in more detail is available in Multimedia Appendix 3.

### Alternative Regulation Approaches

#### Overview

Participants worked on the regulative part during the second half of the focus group discussions. The guiding question was as follows: What could be alternative approaches to regulating medical applications as mobile medical devices? Three themes emerged from these discussions (Textbox 3).

#### Minimal Standards for Medical Apps

Participants emphasized essential minimum standards that medical apps should adhere to ensure safety and harmlessness, including data privacy, user accountability, informed consent, and prevention of biases or discrimination. Experts acknowledged that regulatory requirements should be proportional to the app’s function and data processing. Central to the regulation process is prioritizing the efficacy of the medical app; the efficacy is established through rigorous evaluation procedures.

#### Tools for Regulation

Labels are commonly used in public health and can offer a concise way to communicate information and evaluate app content and user friendliness. To establish a foundational standard for further country-specific development, participants stressed the need for standardized guidelines across geographic regions, exemplified by the medical device regulation in Europe.

#### Barriers to Regulation

The primary concern regarding the law was potential overregulation. Complex approval processes could stifle app innovations. Overregulation might lead to an abundance of specialized “silo apps.” Patients managing multiple health conditions require numerous apps, hindering efficiency and user friendliness. In addition, participants recognized that the app’s usability was contingent on the device, a factor beyond the scope of app regulation.

### Logic Model for the Evaluation and Regulation of Medical Apps

**Figure 3:**
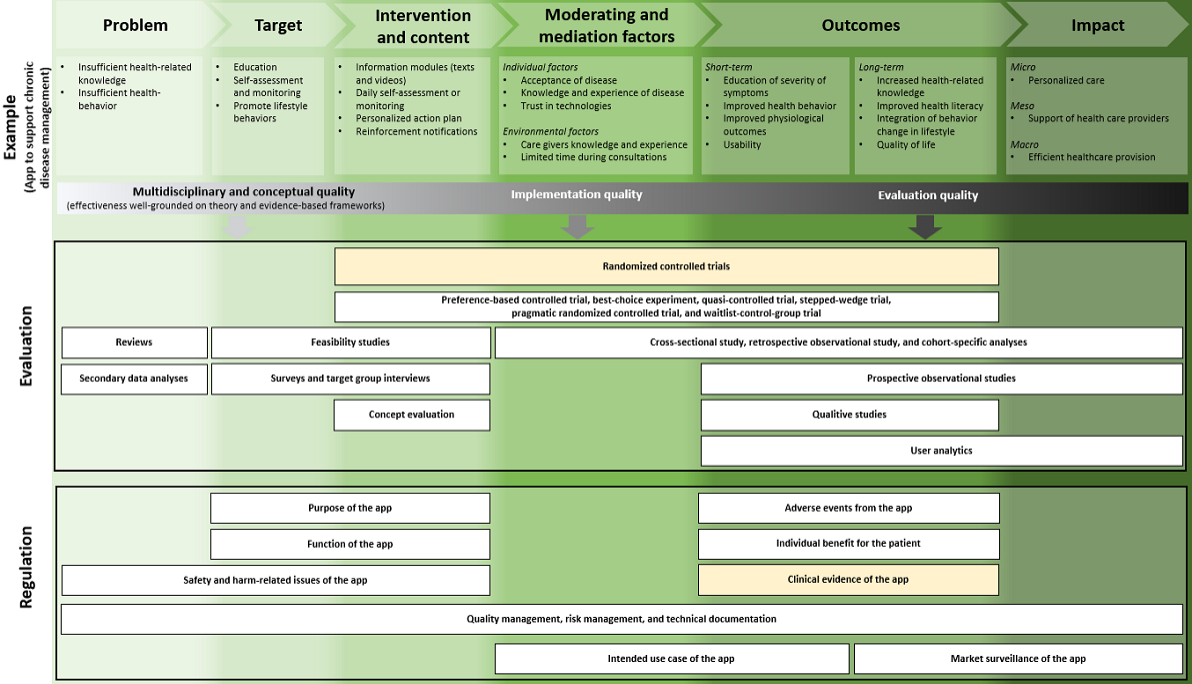
Logic model for evaluating and regulating medical apps through the intervention’s life cycle phases.

On the basis of country-based assessments and the focus group discussions, we developed a logic model highlighting the complexity of medical app evaluation and regulation. A logic model depicts the relationship between a program’s activities and its intended effects [71]. In this study, the logic model illustrates the different stages of app development (read from left to right) along with the various types of evidence and regulation required at these stages [72,73]. The model consists of 3 independent horizontal blocks (Figure 3). The first horizontal block provides an example of a generic medical app. The second horizontal block focuses on evaluation designs for the effectiveness and usability of such apps; the third contains regulatory aspects. Each block is divided into “columns” defined as the general phases of logic models (ie, problem, target, intervention & content, moderating & mediating factors, outcomes, and impact). The boxes on evaluation and regulation are additionally influenced by the conceptual, implementation, and evaluation quality. Apart from the mere analysis of outcomes (as measured through RCTs), it is essential to consider these factors while determining evaluation and regulation approaches for medical apps.

Feasibility studies can be applied to evaluate the medical apps’ target and content. They can include multidisciplinary and participatory designs or theoretical models based on evidence-based frameworks. Machine-learning algorithms and app use modeling from similar data sets can provide additional feasibility analyses [74]. Clinical simulations can be run to test digital interventions in a safe, cost effective, and efficient manner [26]. Alternative trial and study designs that integrate qualitative studies and user analytics (to evaluate the outcomes of the medical app) allow for integrating multiple perspectives and offer efficient approaches to evidence generation. Similarly, the regulation of medical apps should incorporate the app’s life cycle as well. An a priori regulatory concept should be preapplied during the target and content phase of the app development based on the purpose (eg, disease-related) and functionality (eg, documenting, storing, monitoring, data analyzing, or transferring data) of the app. Its potential harm concerning data protection and safety of use (eg, informed consent and appropriateness of data processing) should be best regulated even earlier, that is, during the problem-identification phase. During the outcome phase, relevant regulation is needed in case of adverse events, for patient benefits, and to obtain clinical evidence. The quality and risk management as well as technical documentation should be regulated continuously. Together with the market surveillance after the implementation of the medical app, these procedures will ensure that the application adheres to regulations and fulfills its intended benefits.

“Randomized controlled trials” and “clinical evidence of apps” are highlighted in yellow. This is because most medical apps focus on these methods and outcomes [51]. This is despite the fact that the relevant regulatory framework requires a sufficient evaluation, which does not necessarily have to be an RCT [12]. In addition, our logic model underlines that starting points for RCTs are typically placed too late in the medical app development cycle given that they do not include the two starting phases *problem* and *target*, but instead are placed first in *intervention and content–*defining phases. As such, they are unsuitable for including and assessing all developmental stages.

## Discussion

### Principal Findings

This study explored alternative methods of evaluating and regulating medical apps in an international focus group of public health professionals. The focus group debate highlighted that medical app development could benefit from flexible study designs that can evaluate multiple components simultaneously and independently. While acknowledging the value of RCTs in establishing causal relationships, participants were dissatisfied with RCTs as the “go-to” evaluation method. Instead, they perceived them as a barrier to medical app development. Although participants were aware of various alternative evaluation methods, they found that their application in practice is lacking because the default method remains RCTs.

According to participants, medical app regulation should align with the rapid technological development of medical apps through an agile and iterative evaluation process, which could more appropriately capture the breadth of efficacy and safety outcomes associated with medical apps and potentially reduce the time taken to provide evidence and time taken to launch the app in the market.

The focus group enabled a difficult discussion to challenge the medical dogma of RCTs. Following the global thalidomide tragedy in 1962, an RCT became established as the best practice and is considered a requirement to demonstrate safety and efficacy for drugs entering the drug market [75]. At the time, the scientific community successfully lobbied for stricter evaluation and regulation. In the context of medical apps, regulation is in its infancy. In 2008, the launch of a diagnostic radiology app (MIMvista) at Apple’s World Wide Developers Conference caused a regulatory concern and was arguably the catalyst for medical app regulation worldwide [76]. As scientists, we know the benefits of evaluation. Nevertheless, we now see that RCTs are not apt in the context of the agile nature of digital interventions; viewing RCTs as the status quo stifles innovation in the digital health sector.

The focus group provided an opportunity to share our collective desire for change and collate alternative methods of evaluation and regulation, which are presented in the logic model. This model offers a more dynamic approach to medical app evaluation and regulation. Its structure is designed to incorporate the need for detailed evaluation and regulation of medical apps. The model also encourages researchers to combine alternative study designs to meet their research goals. Alternative study designs mentioned in the focus group include pragmatic RCTs [77,78] such as cohort multiple control randomized studies [79], regression discontinuity designs [80], and registry-based randomized trials [81], which were incorporated into our logic model. To enable sustainable evidence generation, a paradigm shift in the approach to evaluation is needed. Thus, a shift from RCTs as the gold standard to a multimethod, flexible approach that reflects the agility of the medical app market [28] is required. Selected methods should aim to reduce time-to-market access, provide robust evidence on the desired (health) outcomes, and be compatible with the rapid technological development of medical apps.

Finally, it is essential to recognize that digital products constantly evolve, necessitating the ongoing efforts to optimize them retrospectively. Undertaking resource-intensive RCTs to obtain updates on medical apps, such as the German DiGA, may pose significant problems in adherence, increase dropout rate. This may lead to statistically nonsignificant results when analysis is conducted for the whole study population. The high costs and risks of re-evaluating apps using RCTs increase developmental costs, thereby increasing the risk for app developers. This, in turn, increases the probability that apps will not be launched into the market as medical apps but as health and well-being apps, circumventing regulatory approaches. If this happens, overregulation becomes dysregulation.

### Strengths and Limitations

Focus groups have become a frequently used method in health care research. This is due to their usefulness in identifying and analyzing collective opinions and experiences [82,83]. Our study included multinational and multidisciplinary experts, allowing us to collect global perspectives from practitioners and researchers. The participants provided valuable insights on the challenges of using RCTs for medical app evaluation and how alternatives could be applied and integrated into current regulations. Real-world examples were shared as the group included participants who had used RCTs in medical app evaluation or refrained from developing medical apps owing to the challenges discussed in this paper. Finally, our analysis of the 4 most prominently represented countries (ie, Germany, Italy, Canada, and Australia) followed a predefined framework to guarantee comparability between countries [38]. This analysis highlights the lack of a legal obligation to use RCTs to evaluate and regulate medical apps. It suggests that there may be a reflex to use RCTs for evaluation.

Our study comes with certain limitations. Conducting the workshop at an international conference meant that we had no control on recruitment and participant demographics. Despite the global attendance at the conference, this workshop was primarily attended by people living in European and Western countries, with an underrepresentation of participants from the Global South and low- and middle-income countries. Given the international nature of digital health interventions and, in particular, its potential in low- and middle-income countries [84,85], the focus group was not likely to adequately capture the full spectrum of experiences and viewpoints. This limits the generalizability of the findings, particularly given the general underrepresentation of researchers from low- and middle-income countries in discussions about the evaluation criteria of medical apps [86]. With a more diverse participant pool, future research could provide a more comprehensive understanding of challenges and opportunities in medical app regulation. Furthermore, participants differed in their age, academic background, and previous knowledge of this topic. Selection bias, defined as participants choosing to participate based on personal interest, limits the study’s external validity.

Large focus groups, such as our study with 15 participants per group, tend to be challenging to moderate and may not achieve in-depth discussions [82,83]. This was addressed by having experienced focus group moderators who were able to facilitate an open discussion atmosphere. Although approximately 8 participants per focus group are recommended [82,83], larger groups allow for capturing more views in a shorter period. As the goal of the focus group was to gain a broad overview of current views on app evaluation and regulation, large focus groups seemed appropriate.

In addition, the feedback quality was mixed, potentially originating from the self-reported low average experiences with medical app development, evaluation, or regulation (average 1.8, SD 2.7 y; median 1.0, range 0-15 y). Another reason might be a differing understanding of medical apps compared to that of health app. This due to global variations in the definitions [6] and regulation practices associated with health apps. This might have led to an inaccurate participant understanding of medical apps. A 20-minute introductory workshop on RCTs, medical apps, and the regulation approaches for medical apps as mobile medical devices, conceivably cleared up some of the misconceptions.

### Further Research and a First Look at the Desired Future

Medical apps do not necessarily have borders, making this a topic of global interest. Thus, the same challenge occurs in various countries. A focus group discussion with researchers can only be the first step in assessing this topic’s relevance and diverse aspects. Further research needs to develop an evaluation framework that allows different methodological approaches for specific stages during the evaluation of the life span (from development, implementation, and creating broader impact among all stakeholders such as developers, patients, or app prescribers) of a medical app. Simultaneously, this evaluation framework should include regulatory aspects beyond the clinical evidence generated by RCTs.

Establishing the use of evaluation frameworks, such as the proposed logic model, can provide developers and regulators with a scientifically sound set of evaluation criteria that can address the agile and time-efficient development of medical apps while gradually generating evidence. In turn, this will help overcome the misconception of using RCTs as the cultural gold standard for evaluating DiGAs. Furthermore, future research should also focus on other regulatory aspects, such as patient safety and effectiveness, which are vital for the market approval of medical apps. These aspects, however, are not necessarily covered by RCTs. Therefore, future evaluation frameworks must enhance the acceptability of alternative evaluation methods for stakeholders despite the traditional research evidence provided by RCTs.

Medical apps developed by professionals without medical backgrounds can lead to several challenges, including ineffective, unsafe, and difficult-to-use interventions. Therefore, the development of medical applications should be conducted in multiprofessional teams to balance the priority of health outcomes over profitable motives.

Establishing clear regulatory criteria centered on safety, efficacy, and data protection is essential to minimize errors and harm while fully harnessing the potential of medical applications to improve health care delivery. By carefully evaluating hypothetical risks, avoiding unnecessary regulatory burdens (overregulation), and incorporating the potential for learning from manageable errors, innovation, and safety can be balanced in regulating DiGAs. This approach ensures that digital medical apps continue to evolve and improve the quality of health care services while safeguarding the well-being of users.

### Conclusions

While regulations, such as the German DVG or the regulations in Canada and Australia, emphasize the need for comprehensive clinical data, their lack of strict RCT mandates allows for flexibility in evaluating digital health interventions such as medical apps. This flexibility, reinforced by consensus in focus groups, highlights the need for integrating adaptable evaluation techniques into regulatory frameworks. Policy recommendations should include specific guidance regarding the unique considerations of mobile medical devices to bridge the gap between regulatory requirements and evolving scientific methods. Focusing on ongoing assessment, adaptation of evaluation strategies, and balancing patient safety and innovation will create a robust evaluation system for medical apps. This will ultimately empower patients and health care providers while ensuring the safe and effective advancement of digital health technology. Regulatory frameworks should be updated to include guidelines for incorporating real-world data analysis, user engagement studies, and other adaptable evaluation techniques alongside traditional clinical trials.

## Appendix

Multimedia Appendix 1 COREQ (Consolidated Criteria for Reporting Qualitative Research) checklist.

Multimedia Appendix 2 Transcribed flip charts of the focus group discussions.

Multimedia Appendix 3 Glossary of technical terms given during the focus group discussions.

## Abbreviations

DiGA: digital health applications
DVG: Digital Care Act
RCT: randomized controlled trial
SaMD: software as a medical device
TA: thematic analysis
WCPH: World Congress on Public Health
