# Supplementary material for: Challenges and Alternatives to Evaluation Methods and Regulation Approaches for Medical Apps as Mobile Medical Devices: International and Multidisciplinary Focus Group Discussion
Source: J Med Internet Res. 2024 Sep 30;26:e54814. doi: 10.2196/54814 (PMC11474120; doi:10.2196/54814)
Supplement: Multimedia Appendix 1 [file jmir_v26i1e54814_app1.pdf]

Maaß et al. (2024). Challenges and Alternatives to Evaluation Methods and Regulation Approaches for Medical Apps as Mobile Medical Devices: International and Multidisciplinary Focus Group Discussion. JMIR; 26:e54814. https://doi.org/10.2196/54814.

Multimedia Appendix 1: Coreq-Checklist

Topic	Item	Guide Questions/Description	Reported on Page
**Domain 1: Research team and reflexivity**			
Personal characteristics			
Interviewer/facilitator	1	Which author/s conducted the interview or focus group?	6f
Credentials	2	What were the researcher's credentials? E.g. PhD, MD	7
Occupation	3	What was their occupation at the time of the study?	7
Gender	4	Was the researcher male or female?	6f
Experience and training	5	What experience or training did the researcher have?	7
Relationship with participants			
Relationship established	6	Was a relationship established prior to study commencement?	N.A. (No)
Participant knowledge of the interviewer	7	What did the participants know about the researcher? e.g. personal goals, reasons for doing the research	7
Interviewer characteristics	8	What characteristics were reported about the interviewer/facilitator? e.g. Bias, assumptions, reasons and interests in the research topic	7
**Domain 2: Study design**			
Theoretical framework			
Methodological Orientation and Theory	9	What methodological orientation was stated to underpin the study? e.g. grounded theory, discourse analysis, ethnography, phenomenology, content analysis	7ff
Participant selection			
Sampling	10	How were participants selected? e.g. purposive, convenience, consecutive, snowball	6
Method of approach	11	How were participants approached? e.g. face-to-face, telephone, mail, email	6
Sample size	12	How many participants were in the study?	6
Non-participation	13	How many people refused to participate or dropped out? Reasons?	6
Setting			
Setting of data collection	14	Where was the data collected? e.g. home, clinic, workplace	6ff
Presence of non-participants	15	Was anyone else present besides the participants and researchers?	6
Description of sample	16	What are the important characteristics of the sample? e.g. demographic data, date	Table 2
Data collection			
Interview guide	17	Were questions, prompts, guides provided by the authors? Was it pilot tested?	7
Repeat interviews	18	Were repeated interviews carried out? If yes, how many?	N.A. (No)
Audio/visual recording	19	Did the research use audio or visual recording to collect the data?	7
Field notes	20	Were field notes made during and/or after the interview or focus group?	7
Duration	21	What was the duration of the interviews or focus group?	7
Data saturation	22	Was data saturation discussed?	N. A. (No)

Topic	Item	Guide Questions/Description	Reported on Page
**Domain 2: Study design**			
Data collection			
Transcripts returned	23	Were transcripts returned to participants for comment and/or correction?	N.A. (Yes, to those who joined the working group after the workshop)
**Topic**	**Item**	**Guide Questions/Description**	**Reported on Page**
**Domain 3: Analysis and findings**			
Data analysis			
Number of data coders	24	How many data coders coded the data?	7
Description of the coding tree	25	Did authors provide a description of the coding tree?	Textbox 2 & 3
Derivation of themes	26	Were themes identified in advance or derived from the data?	7
Software	27	What software, if applicable, was used to manage the data?	7
Participant checking	28	Did participants provide feedback on the findings?	N. A. (Yes, those who joined the working group after the workshop)
Reporting			
Quotations presented	29	Were participant quotations presented to illustrate the themes/findings? Was each quotation identified? e.g. participant number	Textbox 2 & 3 (only the written statements from the flipcharts)
Data and findings consistent	30	Was there consistency between the data presented and the findings?	9ff
Clarity of major themes	31	Were major themes clearly presented in the findings?	9f
Clarity of minor themes	32	Is there a description of diverse cases or discussion of minor themes?	10ff
