# Supplementary material for: Challenges and Alternatives to Evaluation Methods and Regulation Approaches for Medical Apps as Mobile Medical Devices: International and Multidisciplinary Focus Group Discussion
Source: J Med Internet Res. 2024 Sep 30;26:e54814. doi: 10.2196/54814 (PMC11474120; doi:10.2196/54814)
Supplement: Multimedia Appendix 2 [file jmir_v26i1e54814_app2.pdf]

Multimedia Appendix 2: Transcribed flipcharts of the focus group discussions

Group 1

Evaluation

- Step-wedged designs
- Participatory research (UX-perspective)
- Shortcut evaluation when we know one aspect works (puzzle intervention)
- Prototype checklist of evaluation
- Speed of developments vs. RCT duration
- Pragmatic RCTs
- Real-world evidence (e.g., hospital data, cohort data set)
- What component of the intervention is effective?
    - Output vs. outcome evaluation

Regulation

- Using labels, also rate accessibility and not only on health labels
- Regulations proportional to what app does
- No overregulation
- silo apps → if a chronically ill person needs 20 apps, it is not feasible and very expensive

Group 2

Evaluation

- RCTs are stronger in statistics but not realistic
    - People are not always happy with the randomization & might drop out if clustered in the wrong intervention (prefer app or standard care)
- Preference-based trials
    - Not randomized, participants choose the intervention they prefer most
    - Shorter in recruitment
    - More pragmatically approach
    - Better data quality due to less drop-out (no forcing to use the intervention or not) ("for our RCT, only 20% of intervention participants used the app after the login screen. So, if we would have kept all 100% intervention-participants for the data analysis, there would have been no results. However, for those 20%, there were significant differences compared to standard care")
    - Possibility to embed these trials in the introduction/implementation of the intervention in the healthcare system/app store
    - "participants have control over their treatment"
- Can you embed preference with an RCT?

  - Best choice experiment after randomization
  - Only if you do not lose people in the initial stage
  - Or at least ask people what their preference would have been
- Stepped wedge trial – make it available to different cohorts in a staggered way
  - Recruitment until (e.g.) 30 people per arm then have these use the intervention/control already (cohort 1) while recruiting 30 more per arm (cohort 2), etc.
  - Shorter study duration as some data is collected already during recruitment for more participants
- Testing app algorhythm on another disease database to see if algorithm would fit the target population & their disease (take sociodemographic data from database for testing prognostic behavior with app)
- Synthetic data generated from a dataset with a similar distribution as we'll expect for the app's target population → use this data to model the use of the app
- Effectiveness trials tend to produce too few engagement à if this is the case, do user interviews to understand engagement
- Need trials embedded in practice/roll out of service
  - People join the service first & invite them to take part in the research to use their routinely collected health data to evaluate app effectiveness (app evaluates itself as a before-after style)
- Use google analytics to evaluate use
  - is there available data to help evaluate effectiveness via sensors/google/ect?
- "the device is equally important to be considered as the app"

Regulation

-

Group 3

Evaluation

- Which outcomes?
  - Multiple outcomes vs. standardized outcomes
  - Which outcomes for whom?
  - Qualitative pre-study
  - Level of interaction → how often do you use it?
- Sample size, power sample, bias, level of causality, purity of data
  - Comparability → new intervention or existing intervention → against what to we compare?
- Hawthorne-effect
- Traditional vs. implementation outcomes produces different types of data

Regulation

- Privacy of data
- Accountability → informed consent
- Standardized regulation → experience in Europe
    - Minimal requirements
- Efficacy
- Do no harm interventions
- Country examples for regulations
- No discrimination
