# Supplementary material for: Challenges and Alternatives to Evaluation Methods and Regulation Approaches for Medical Apps as Mobile Medical Devices: International and Multidisciplinary Focus Group Discussion
Source: J Med Internet Res. 2024 Sep 30;26:e54814. doi: 10.2196/54814 (PMC11474120; doi:10.2196/54814)
Supplement: Multimedia Appendix 3 [file jmir_v26i1e54814_app3.pdf]

Maaß et al. (2024). Challenges and Alternatives to Evaluation Methods and Regulation Approaches for Medical Apps as Mobile Medical Devices: International and Multidisciplinary Focus Group Discussion. JMIR; 26:e54814. https://doi.org/10.2196/54814.

Multimedia Appendix 3: Glossary of technical terms given during the focus group discussions

Best choice experiment after randomization: A preference-based controlled trial in which participants are asked to choose between different interventions after being randomly assigned to a treatment group.

Hawthorne effect: The tendency for people to modify their behavior simply because they are being observed.

Overregulation: Overregulation in medical apps refers to the excessive or burdensome regulation of these apps. This can include regulations that are poorly designed, outdated, or not based on evidence. Overregulation can stifle innovation and make it difficult for digital health apps to enter the market or be effective.

Participatory research: Research that involves the active participation of those who will be affected by the research findings.

Pragmatic randomized controlled trials (RCTs) / Real-world RCTs: A type of RCTs designed to be more representative of real-world settings. Pragmatic RCTs often use less restrictive eligibility criteria and allow for more flexibility in the delivery of the intervention.

Preference-based controlled trials: A type of controlled trial in which some participants are asked to express their preferences for different interventions before being assigned to a treatment group.

Quasi-controlled trial/Quasi-experiment: A quasi-controlled trial is a study that aims to evaluate the effects of an intervention on an outcome but does not use randomization to assign participants to the intervention or control group.

Shortcut evaluation: A simplified approach to evaluation that is used when a more rigorous evaluation is not feasible or necessary.

Stepped wedge trials: A type of RCT in which groups of participants are randomly assigned to receive the intervention at different time points. This can help reduce the trial's cost and make it more feasible to conduct in real-world settings.

Traditional RCTs: A type of clinical trial in which participants are randomly assigned to either a treatment group (which receives the intervention being studied) or a control group (which does not receive the intervention). RCTs are considered the gold standard for evaluating the effectiveness of medical interventions, as they minimize bias and allow for causal inferences to be drawn.

User experience (UX) (for digital health apps): How users interact with and experience these apps. UX encompasses all aspects of the app, from its design and functionality to its content and accessibility.

Waitlist-control-group design: A research design in which the control group is placed on a waitlist to receive the intervention after the treatment group has completed the study.
